# Dutch translation and linguistic validation of the U.S. National Cancer Institute’s Patient-Reported Outcomes version of the Common Terminology Criteria for Adverse Events (PRO-CTCAE™)

**DOI:** 10.1186/s41687-020-00249-y

**Published:** 2020-10-06

**Authors:** Evalien Veldhuijzen, Iris Walraven, Sandra A. Mitchell, Elizabeth Yohe Moore, Shawn M. McKown, Matthew Lauritzen, Katherine J. Kim, José S. A. Belderbos, Neil K. Aaronson

**Affiliations:** 1 Department of Radiation Oncology, The Netherlands Cancer Institute, Antoni van Leeuwenhoek, Amsterdam, the Netherlands; 2 Department for Health Evidence, Radboud University Medical Center, Nijmegen, the Netherlands; 3 Outcomes Research Branch, Division of Cancer Control and Population Sciences, National Cancer Institute, Bethesda, USA; 4 RWS Life Sciences, East Hartford, USA; 5 Genentech – a member of the Roche Group, South San Francisco, USA; 6 Division of Psychosocial Research and Epidemiology, The Netherlands Cancer Institute, Plesmanlaan 121, 1066 CX Amsterdam, The Netherlands

**Keywords:** PRO-CTCAE, Patient-reported outcomes, Symptomatic adverse events, Translation, Linguistic validation, Cancer

## Abstract

**Background:**

The U.S. National Cancer Institute’s Patient-Reported Outcomes version of the Common Terminology Criteria for Adverse Events (PRO-CTCAE™) is a library of items for assessing symptomatic adverse events by patient self-report in oncology trials. The aim of this multi-site study was to generate and linguistically validate a Dutch language version of the U.S. PRO-CTCAE for use in the Netherlands and Dutch-speaking Belgium.

**Methods:**

All 124 items in the PRO-CTCAE item library were translated into Dutch using established translation procedures, including dual forward translations, reconciliation, back-translation, reconciliation of the source with the back-translation, and expert reviews. Harmonization of the translation for use in both the Netherlands and Belgium was achieved via an iterative review process in which the translations were discussed and reconciled by consensus of PRO experts, clinicians and bilingual Dutch translators. The translated PRO-CTCAE™ items were completed by a geographically-diverse sample of Dutch speaking patients from the Netherlands (*n* = 40) and Belgium (*n* = 60), and who were currently receiving or who had recently completed cancer-directed therapy. Patients were diverse with respect to age, sex, educational attainment, and cancer diagnosis. Cognitive debriefing, using a semi-structured interview guide, probed for comprehension and clarity of PRO-CTCAE symptom terms, attributes (e.g. frequency, severity, interference), response choices, and understanding of ‘at its worst’ and ‘in the last 7 days’. Items for which the patient data indicated possible difficulties were considered for revision.

**Results:**

Three items underwent minor phrasing revision and retesting was not deemed necessary. The symptom term for stretch marks was poorly understood by 12.5% of participants, and this item was revised to include parenthetical phrasing. It was retested with 10 participants from Belgium (*n* = 5) and the Netherlands (*n* = 5) and demonstrated acceptable comprehension.

**Conclusions:**

The Dutch language version of PRO-CTCAE has been successfully developed and linguistically validated for use in oncology studies in the Netherlands and Dutch-speaking Belgium. Extending the availability of NCI PRO-CTCAE in languages beyond English increases international consistency in the capture of Patient-Reported outcomes in patients participating in cancer clinical trials.

## Background

Reporting of adverse events (AE) is an essential part of oncology trials and is commonly performed by clinicians using the Common Terminology Criteria for Adverse Events (CTCAE). There is evidence that precision and reliability in capturing patient’s experiences of symptomatic AEs (e.g. pain, fatigue, and nausea) can be improved through the use of self-report data [1–4]. This has led to the development of symptom reporting measures that rely on direct feedback from patients. These Patient-Reported outcome measures (PROMs) exhibit improved validity and reliability when compared to clinician-reported outcomes [2, 3]. Moreover, the use of PROMs for monitoring side effects during cancer treatment may improve both quality of life and overall survival [5, 6]. These observations have led to a growing support for the development and use of PROMs for symptomatic AE reporting [7–10] .

The U.S. National Cancer Institute (NCI) developed a CTCAE-based PROM for descriptive patient reporting of symptomatic side effects, the Patient-Reported Outcomes version of the Common Terminology Criteria for Adverse Events (PRO-CTCAE) [11]. PRO-CTCAE consists of 124 items assessing 78 symptomatic toxicities drawn from CTCAE that were identified as amenable to patient self-report. PRO-CTCAE items cover a wide range of symptomatic adverse events including events in the following categories; oral, gastrointestinal, respiratory, circulatory, cutaneous, neurological, visual, attention, pain, sleep/wake, mood, genitourinary, sexual and other. For each of these symptomatic AEs, PROM items are available reflecting the attributes of frequency, severity and/or interference with usual or daily activities [12]. For any given AE, the PRO-CTCAE contains 1 to 3 attributes depending on the CTCAE criteria and nature of that AE. PRO-CTCAE responses are scored from 0 to 4 (or 0/1 for absent/present). Each PRO-CTCAE item includes a plain language description of the symptomatic AE, together with the attribute of interest. The standard recall period for the CTCAE items is ‘the past 7 days’. In any given trial, investigators select a subset of these items for evaluation based on study hypotheses, prior research, and knowledge of the anticipated regimen-related toxicities. The PRO-CTCAE item library offers a systematic yet flexible approach to capture symptomatic adverse events.

The measurement properties of PRO-CTCAE have been comprehensively evaluated in English-speaking patients [13, 14] and PRO-CTCAE has been translated into more than two dozen languages, with additional languages currently in development [15–18]. Extending the availability of the NCI PRO-CTCAE™ beyond the English language increases international consistency in symptomatic AE reporting by patients participating in cancer clinical trials. The aim of the current study was to translate PRO-CTCAE into Dutch and linguistically validate PRO-CTCAE-Dutch for use in the Netherlands and in Dutch-speaking Belgium.

## Materials and methods

To ensure a robust methodology and interpretation of study results, a, research collaboration was established between a Dutch steering group, professional translators and representatives from the U.S. NCI PRO-CTCAE study group. The methodology used in this study consisted of forward-backward translation followed by cognitive debriefing, and is consistent with guidelines recommended by the International Society for Pharmacoeconomic and Outcomes Research (ISPOR) guidelines for translation and cultural adaptation of PRO instruments [19].

### Translation

The complete English language version of the PRO-CTCAE item library was translated into Dutch using a forward-backward translation method. First, two native Dutch speakers fluent in English translated all items from English to Dutch (forward translation). Any differences in item translation were discussed and reconciled by a third native Dutch speaker, resulting in a single, reconciled translation. Next, the reconciled forward translation was back-translated into English by a native English speaker fluent in Dutch, and this back translation was compared with the original English language version to identify any possible discrepancies requiring further attention and another round of forward and back translation. Harmonization of the translation for use in both the Netherlands and Belgium was achieved via an iterative review process in which the translations were discussed and reconciled by a consensus of clinical experts and bilingual Dutch and Belgian translators. Items that were identified as potentially problematic during the translation process were flagged for special attention during subsequent cognitive interviewing. All steps in the translation process were documented in writing and reviewed by the research team for quality control.

### Patient sample and setting

A heterogeneous sample of cancer patients took part in the study. Study participants were recruited from a variety of hospitals and patient advocacy and support groups in Belgium and from two hospitals in the Netherlands (Antoni van Leeuwenhoek Hospital (Amsterdam) and Institute Verbeeten (Tilburg). Efforts were made to accrue a range of participants in terms of cancer site, treatment type, sex, years of education, performance status (in the Belgian sample) and province of residence. Individuals were eligible for the study if they: were currently undergoing cancer treatment or had completed treatment in the past 6 months; were 18 years of age or older; and were Dutch speakers (as spoken in the Netherlands or Belgium) with sufficient command of written and spoken Dutch to complete questionnaires and participate in the cognitive interviews. Individuals were excluded if they exhibited serious cognitive or psychiatric problems as judged by their healthcare provider. All participants provided written informed consent. All data were collected during a single appointment between the interviewer and the study participant.

### Cognitive interviewing procedures

Interviews were conducted in a private area of the clinic and consisted of two parts: (a) administration of a PRO-CTCAE survey comprised of a subset of PRO-CTCAE items; and (b) a semi-scripted debriefing interview with cognitive probing of comprehension, clarity and ease of judgement. All participants were first asked to complete a short demographic questionnaire, followed by a customized PRO-CTCAE survey. To minimize respondent burden, all participants completed 19 PRO-CTCAE items that were selected a priori on the basis of their prevalence across cancer treatment types and disease sites [13, 20]. These 19 core items addressed the following symptom terms: shortness of breath, concentration, memory, arm or leg swelling, numbness and tingling, insomnia, fatigue, rash, pain, headache, anxiety, discouraged, sad, decreased appetite, nausea, vomiting, constipation, diarrhea and mouth or throat sores. Items reflecting a subset of the remaining 59 PRO-CTCAE symptom terms were included in each customized survey.

Eight different PRO-CTCAE surveys (four for men and four for women) were generated to ensure that all PRO-CTCAE items were distributed evenly across study participants, with stratification by sex and cancer type. Following this distribution, items reflecting 14 of the 78 PRO-CTCAE symptom terms were evaluated by 100% of the sample, and an additional four symptom terms were evaluated by 50% of participants. Of the remaining symptom terms, the items that assess 54 PRO-CTCAE symptom terms were evaluated by 25% of the sample, and items that capture the six gender-specific PRO-CTCAE symptom terms were evaluated by 12.5% of participants (see Supplementary Table 1 for more details about the number of respondents debriefed and the proportion experiencing comprehension difficulties by PRO-CTCAE item).

While completing the PRO-CTCAE survey, participants were asked to tick a box if they found questions to be difficult to understand, had difficulty selecting a response, or if they had comments about the specific phrasing of the PRO-CTCAE-Dutch questions or found the phrasing upsetting. Additionally, the interviewer noted any spontaneous observations or comments made by the participants that might indicate comprehension problems or other difficulties experienced as they completed their surveys. Surveys were independently completed on paper by the participant. Respondents also had the option to have the PRO-CTCAE items read to them verbatim if their physical condition limited their ability to complete the survey independently. Participants were asked to indicate those PRO-CTCAE questions that they found difficult to comprehend and those for which they had difficulty selecting a response.

After completing the PRO-CTCAE survey, participants underwent a semi-structured cognitive interview that included a think-aloud paraphrase task to confirm understanding of the PRO-CTCAE symptom terms, the attributes, the response choices, and understanding of ‘at its worst’. Probes used to elicit the participants comments on general comprehension included; ‘What was your general impression of the questions?’ and, ‘Were there issues that made it difficult to answer any of the questions?’. The attribute probes included questions such as: ‘What does the word ‘INTERFERE’ mean in this [example] question’ or ‘What does the phrase SEVERITY at its WORST mean in this [example] question?”. The example symptomatic AEs that were used for these questions included fatigue, nausea and pain. Symptom terms that were identified by the study team during the translation process as possibly being difficult to comprehend or judge when posed in Dutch, and, items that the individual participant had flagged as being difficult to answer received special attention during the debriefing process. Several probes were used to gain fuller insight into participants’ understanding of the questionnaire content. These included questions such as: ‘What does this symptom mean to you?’, and ‘Are there other terms that you would use to describe this symptom?’. All interviews were carried out by trained interviewers with a background in health sciences or translation, and experience in conducting interviews in medical settings. All comments, feedback and interviewers’ field notes were collated in a testing report.

### Data analysis

As these linguistic validation data are qualitative in nature, the analysis followed an established methodology for cognitive interviewing [21, 22]. All participant responses were summarized by the interviewer and documented in a testing report, including participants’ comments and any suggestions for phrasing revision, when deemed necessary. A bilingual investigator at the Netherlands Cancer Institute reviewed the summaries of 20% of the interviews for quality control and to ensure concurrence on the recommended actions.

We evaluated respondents’ general impressions of the PRO-CTCAE symptom terms and item structures, as well as their feedback on comprehension, clarity and ease of judgement of the PRO-CTCAE items. We specifically probed comprehension of the PRO-CTCAE symptom terms, the attributes, clarity of the phrasing ‘at its worst’, and the response options. Similar responses were grouped and then categorized into the themes; comprehension/understanding, clarity of meaning, interpretation of the symptom term itself and the symptom attribute (i.e., frequency, severity, and interference), ease of recalling the information needed to respond, and the extent to which personal responses fit the available response options.

Cognitive debriefing results were summarized by the interviewer on an item-by-item basis and included a recommended course of action for each item. Recommendations for action included review of the findings with members of the study team and the linguistic consultants. The possible courses of action were: a) no change necessary (no participants reported difficulties with clarity or understanding of the questionnaire item; b) a change may be necessary (up to 20% of the participants indicated difficulties with clarity or understanding); or c) a change is definitely necessary (20% or more of the participants indicated having difficulty with clarity or understanding). All items that were flagged by at least one participant as difficult to understand or answer were reviewed and discussed within the local study team and, when deemed necessary, with the US-based PRO-CTCAE developers. When modifying an item, the documentation of the item history was reviewed, including the field notes from the interviewers, in an attempt to find a better translation option. Items that required substantial modification were retested in a second round of cognitive interviews using similar interviewing procedures and probes.

## Results

### Translation

There was full agreement on the two forward and the backward translations for 21 and 71 PRO-CTCAE symptomatic toxicities, respectively. Fifty-seven instances of a difference between the Dutch and Belgian language versions were identified in the forward translations. These were reconciled to create a universal harmonized Dutch translation. In the back translation, there were subtle phrasing differences for symptom terms compared to the English source. These differences were discussed among the study team, and the Dutch phrasing of these items was adjusted to balance respondent comprehension with fidelity to the precise clinical meaning of a specific symptomatic AE.

### Sample characteristics

Between May 2017 and January 2018, a total of 105 participants were recruited into the study (Round 1: *n* = 100; Round 2: *n* = 5). In Round 2, a subset of Round 1 participants from Belgium were re-utilized (*n* = 5), while new participants (*n* = 5) were recruited in the Netherlands for Round 2. Participants were diverse with respect to age, educational attainment, performance status (recorded only in the Belgian sample), cancer diagnosis and treatment received (Table 1). All geographical regions (provinces) within the Netherlands and Dutch-speaking Belgium were represented in the sample. In round 1, 51% of the participants was female, the mean age was 59 years (range 28–85 years) and the average number of years of formal education was 14.4 years (SD = 2.3 years; range = 9–18 years). Nine cancer sites were represented in the sample, and the treatment modalities received by participants were diverse.

**Table 1:** Demographic and clinical characteristics Round 1(*n* = 100) and Round 2 (*n* = 10)

Participant characteristics	Round 1 *n* = 100	Round 2 *n* = 10^a^
Age		
Mean (SD)	59.3 years (±12.1)	63,4 (±8.5)
Range in years	28–85	51–80
% > 65 years of age	34%	30%
Sex n (%)		
Female	51 (51)	4 (40)
Male	57 (57)	6 (60)
Education		
Mean years (SD)	14.4 (2.3)	12.0 (1,6)
% lower than high school: (or nearest equivalent)	7	20
% high school or higher: (or nearest equivalent)	93	50
Missing (%)	0	30
Type of treatment n (%)^b^		
Combined Chemo-radiotherapy	36 (36)	5 (50)
Chemotherapy	26 (26)	5 (50)
Radiotherapy	39 (39)	2 (20)
Hormonal therapy	13 (13)	1 (10)
Part of clinical trial	5 (5)	0 (0)
Immune therapy	10 (10)	0 (0)
Molecularly targeted therapy	8 (8)	1 (10)
Cancer site n (%)		
Breast	24 (24)	2 (20)
Hematological	22 (22)	3 (30)
Urological	14 (14)	2 (20)
Lung	13 (13)	0 (0)
Gynecological	11 (11)	2 (20)
Head and neck	10 (10)	1 (10)
Gastrointestinal	8 (8)	0 (0)
Brain	4 (4)	0 (0)
Skin	2 (2)	0 (0)
Performance status^c^		
Karnofsky Performance Scale Index		
90–100	17 (17)	2 (20)
60–89	20 (20)	3 (30)
< 60	3 (3)	0 (0)
Missing %	60 (60)	5 (50)
Country n (%)		
The Netherlands	60 (60)	5 (50)
Dutch-speaking Belgium	40 (40)	5 (50)

^a^ In Round 2, a subset of participants (*n* = 5) from Belgium were re-interviewed and five new participants were recruited in the Netherlands

^b^Some participants received more than one treatment type

^c^ Performance status was only captured in the Belgian sample

### General impression of the PRO-CTCAE items structures

A majority of study participants in Round 1 (97/100) were able to complete the PRO-CTCAE survey without any assistance (97/100) Three participants requested interviewer assistance to complete their PRO-CTCAE survey, due to physical discomfort or physical limitations (e.g. pain, being bedridden). Most of the participants’ general comments (*n* = 14) about the general features of the PRO-CTCAE centered on the branching structure of the questionnaire. That is, some respondents found the PRO-CTCAE symptom terms that are assessed using more than one attribute (i.e. those AEs with a frequency, severity and/or interference question) to be somewhat confusing because they were not experiencing the symptom. These participants indicated that they should be instructed to skip the follow-up questions or that a ‘not applicable’ response option should be added. A small group of respondents (*n* = 7) indicated that they did not like responding to items that are conditional in nature (e.g., where a question about the frequency of a symptom is contingent on experiencing the symptom at all). As one patient stated: ´*This question assumes I experience the symptom, but I don’t*´.

Five participants were unclear about whether to report symptoms that might not be specifically related to their cancer treatment experience. For example, they were unsure whether they should report having memory loss or problems with low libido, if they believed it was due to aging rather than to their cancer or its treatment.

Four participants indicated that they would have liked to have had an opportunity to elaborate on their response to the PRO-CTCAE structured questions. For example, two participants indicated that they would have liked to have been able to report that they were taking medication or had taken other steps to alleviate their symptoms. Four participants suggested additional symptoms that they would have liked to have been able to report, including dry eyes (*n* = 1), dry and cracked lips (*n* = 1), mucus formation (*n* = 1), and loss of sense of smell (*n* = 1).

### PRO-CTCAE symptom terms

Forty-eight of the 78 symptom terms were rated as difficult to answer by one or more of the Dutch speaking participants (ranging from 3% to 16%). All of these items were discussed with the study team (Table 2).

**Table 2:** PRO-CTCAE symptom terms presenting comprehension difficulties across 2 rounds of cognitive interviews

Source of Difficulty	Proportions with difficulty Round1 n (%)	PRO-CTCAE Symptom term	Examples of difficulties experienced by participants in Round 1	Decisions after Round 1	Decisions after Round 2
Comprehension	3/24(12.5)	Hives *Netelroos*	Participant was not sure he understood what was meant by ‘hives’	No suitable alternative term; no revision	Not applicable
	3/24 (12.5)	Stretch marks *Striemen*	Participant comments suggested that the Dutch terminology chosen for ‘*stretch marks*’ was not well understood	Revised to include parenthetical phrasing and retested	Well comprehended; revised phrasing retained
	2/21 (10)	Nail ridging *Geribbelde nagels*	Participant was confused with the description of bumps and ridges; they did not understand that this could occur with finger or toenails	No suitable alternative term; no revision	Not applicable
	1/24 (4)	Gas *Winderigheid*	Participant did not understand the specific phrasing for ‘flatulence’, although they were able to comprehend the meaning of the item overall	No revision	Not applicable
	6/90 (6.6)	Mouth and throat sores *Zweertjes in de mond/keel (aften)*	Participant stated that it was not clear whether this question was also about blisters in the mouth.	No suitable alternative term; no revision	Not applicable
	1/21 (4.8)	Ringing in ears *Oorsuizen*	One participant did not understand the phrasing chosen for ´ringing in ears’.	Most participants comprehended this item well; no suitable alternative; no revision	Not applicable
Clarity of phrasing (intent or meaning of item unclear)	1/24 (4.2)	Visual floaters *Zwevende vlekjes of sliertjes die voor uw ogen bewegen*	One participant misinterpreted ‘visual floaters’ as ‘blurred vision’	Most participants were clear about the meaning of this item; no revision	Not applicable
	1/21 (4.8)	Urinary urgency *Urinaire aandrang*	One participant thought that the phrasing ‘all of a sudden’ implied that urinary urgency was unexpected; this participant was experiencing chronic urinary urgency	Most participants were clear about the meaning of this item; no revision.	Not applicable
	2/24 (8.3)	Decreased libido *Verminderd libido*	Participant had a decreased sexual interest, but attributed this to his age	Most participants were clear about the meaning of this item; no revision	Not applicable
	2/24 (8.3)	Unable to achieve orgasm *Uitgesteld orgasme*	The Dutch phrasing of ‘*unable*’, combined with the response choice “*no*’, resulted in misinterpretation due to double negative	Item revised to improve clarity: In the last 7 days, was it IMPOSSIBLE TO HAVE AN ORGASM OR CLIMAX?	Not applicable
	4/21(19)	Increased sweating *Toegenomen transpiratie*	Participant found it difficult to differentiate when sweating is due to hot flushes and when not	No suitable alternative; no revision	Not applicable
	7/90 (7.8)	Anxious *Angst*	Participant was not sure whether the items about anxiety refer to general anxiety or cancer- related anxiety	No suitable alternative; no revision	Not applicable
	3/70 (4.2)	Rash *Huiduitslag*	Participant comments indicated confusion about what types of rash were included in this item	Item revised to improve clarity: In the last 7 days, did you have any form of RASH?	Not applicable
	3/19 (15.8)	Achieve and maintain erection *Erectie krijgen en behouden*	Phrasing of this item in the source language made phrasing in Dutch confusing and unclear (i.e., “*severity of your difficulty*” could not be well translated).	Revised item: In the last 7 days, how SERIOUS were your PROBLEMS WITH GETTING OR KEEPING AN ERECTION at their WORST?	Not applicable
Ease of judgement, recall and response	1/21(5.)	Pain and swelling at injection site *Pijn en zwelling bij injectieplek*	Participant stated that this question asks about two things, and is therefore difficult to answer	Item generally well comprehended; no revision.	Not applicable
	1/21 (4.7)	Body odor *Lichaamsgeur*	Participant stated that this is difficult to judge about yourself	Item generally well comprehended; no revision	Not applicable
	2/39 (9.5)	Vaginal dryness *Vaginale droogheid*	Participant found this difficult to judge because she was not sexually active	Item generally well comprehended; no revision	Not applicable

Ultimately four items required revision (Table 2). For the item “rash” (*huiduitslag*), three participants did not understand that the question referred to any type of skin rash (and not only to a specific type). This resulted in a slight rephrasing of the PRO-CTCAE-Dutch item for rash to refer explicitly to ‘any form of rash’ (*enige vorm van huiduitslag*). Given the very minor nature of this revision, retesting of this item was not deemed necessary.

In Dutch, the word for stretch marks (*striemen*) can be interpreted in different ways. This led to misunderstanding by 3 of 24 Dutch speaking participants. For example, one participant asked whether this also refers to marks left by a tight belt. For most Dutch participants, the Dutch word *striae* better matched the intended meaning of the term stretch marks, this item was therefore revised to replace the phrasing stretch marks with *striae* and retested with 10 participants (5 in Belgium and 5 in the Netherlands). However, while Dutch participants were more familiar with the term *striae*, most Belgian participants (*n* = 3) were not. Two of the Belgian participants noted that the term *striae* was more likely to be used by individuals with higher educational attainment. Therefore, in order to promote comprehension by all patients, it was decided to keep the term *striemen* while adding the term *striae* in parentheses to the universal Dutch translation.

Finally, two PRO-CTCAE items, difficulty achieving and maintaining erection, and inability to have an orgasm, underwent minor rephrasing after Round 1. For the item concerning achieving and maintaining an erection, use of the Dutch word ‘*erg’* (used to translate the English term ‘severity’) resulted in 3 of 19 (15.8%) participants interpreting the question as meaning the extent to which one was bothered by having erectile problems, rather than how severe the problem was, as originally intended. This led to a minor revision of the item wording (see Table 2). In addition, two of 24 (8.3%) participants reported difficulty in understanding the item regarding inability to have an orgasm. In Dutch, the phrasing of the question, in the context of the response categories, resulted in a double negative. Replacing the phrase ‘*were you unable’* (‘*was u niet in staat’*) with ‘*was it impossible’* (‘*was het onmogelijk’*) in the stem of the item resolved the comprehension difficulty without having to rephrase the standard response options. Since the nature of this revision was not based on comprehension of the symptom term itself, the study team did not believe that this rephrasing warranted retesting.

### Comprehension of PRO-CTCAE attributes, response options and concept of ‘at its worst’

While the concept ‘severity’ was generally well understood, 7 of the 100 participants did experience some comprehension difficulties with this attribute. For example, one participant pointed out that the meaning of severity may vary, depending on the extent to which one has experienced and perhaps adjusted to a symptom. Similarly, the concept ‘frequency’ was also generally well understood by most participants, although some participants (*n* = 13) all from the Belgian cohort, preferred a somewhat more colloquial word (*dikwijls*) over the original Dutch translation (*vaak)* to indicate ‘often’ as a frequency response choice. However, as all participants understood the harmonized Dutch phrasing, no change was made to these two elements of the translation.

Lastly, the concept ‘interference’ was also well understood by a majority of participants. However, a few participants (*n* = 6) in the Dutch cohort indicated that the chosen Dutch word for ‘interference’ (‘*belemmeren*’), was quite strong, and that it was not ‘in their character’ to allow symptoms to interfere with their lives. Two Belgian participants indicated they associated the translation of ‘interference’ with the concept of being unable to perform that activity altogether (‘*tegenhouden’*). Since this lack of clarity was reported by only two participants in the sample and because no better alternative phrasing could be identified for the attribute of interference, the original translated terminology was retained.

Participants were also queried about several reoccurring elements in the questionnaire, including the recall period, and the phrase ‘at its worst’. The recall of ‘the last 7 days’ was well understood, but some participants (*n* = 9/100; 1 in Belgium and 8 in the Netherlands) found it challenging to judge when a symptom was ‘at its worst’ (‘*op het slechtste moment*’). As one participant put it: ‘*it is difficult to know because I experience the symptom constantly’*. Some of these participants also noted that it was easier for them to rate the overall severity during the past week than to judge the severity at its worst during the past week.

The PRO-CTCAE response options were generally well understood. Only 4 participants (2 Belgian and 2 Dutch) indicated that they would prefer to respond using a 1–10 numerical scale. This is a response scale metric that is commonly used in the educational system and in some other Dutch-language surveys.

## Discussion

In this study we used a rigorous and well-established methodology [21, 22] to generate a harmonized Dutch language version of the entire PRO-CTCAE item library for use in both the Netherlands and in Dutch-speaking Belgium. This methodology is line with both that used in the development of the original, English language version of the PRO-CTCAE and other PRO-CTCAE translation efforts that have been carried out in Danish, German, Spanish, Japanese and Korean [14–17, 23, 24]. More information about the PRO-CTCAE and permission to use this Dutch language version of the instrument can be obtained at http://healthcaredelivery.cancer.gov/pro-ctcae.

The harmonized forward and backward translations showed consistency with the meaning of the source PRO-CTCAE English language material. Overall, the results indicated that the translated version of the questionnaire was well understood by participants with varying levels of education, different cancer diagnoses and stage of disease, and different treatment experiences. Our results are also similar to those of other PRO translation efforts, such as the Dutch version of the Patient Reported Outcomes Measurement Information System (PROMIS) where, for example, similar wording has been used for the Dutch translation of the term ´interference´ (*belemmering*) as well as ´how much´ (*in welke mate*) [25].

Four PRO-CTCAE-Dutch symptoms terms required rephrasing based on participant feedback. The PRO-CTCAE item regarding stretchmarks, which required an elaboration in parentheses, was also identified as somewhat problematic in the Danish linguistic validation study of the PRO-CTCAE [15]. This suggests that the misinterpretation by some participants might not solely be an issue of language or culture but might be due to participants being unaware that this symptom is particularly relevant in the context of cancer treatment and not just pregnancy or weight gain.

A small group of participants found reporting the severity of symptoms somewhat difficult because judgement of severity may vary, depending on the extent to which one has experienced and perhaps adjusted to a symptom. This “response shift” phenomenon has been discussed extensively in the literature on health status and quality of life assessment in oncology settings [26, 27].

A few participants stated that they would have liked to have been able to report additional symptoms. While this was not explicitly tested in this study, a PRO-CTCAE free text question is included as part of the NCI PRO-CTCAE item library, and we would encourage its use in practice [28, 29].

Approximately 13% of study participants experienced some difficulty in interpreting the phrase ´*at its worst*´. A similar issue was also reported in the cognitive interviewing study that was part of the development of the original U.S. English version of the PRO-CTCAE, and was also observed in the PRO-CTCAE-Japanese linguistic validation study [14, 23]. Specifically, a minority of patients may focus on average severity of their symptoms rather than on the severity ´at its worst´. Formatting conventions for PRO-CTCAE include capitalization of this phrasing to emphasize to respondents that they should focus on worst rather than on average severity [14]. In addition, there is psychometric evidence that 1 week recall of severity at its worst corresponds best to the worst daily report, compared to longer recall periods of 2, 3 or 4 weeks [30].

A larger group of participants suggested that when a given symptom is not experienced that follow-up questions be avoided or that a ´not applicable´ option be added. However, we would note that this issue is relevant only when the PRO-CTCAE is administered as a paper questionnaire. The PRO-CTCAE Measurement System was developed and tested with a conditional branching logic that helps to reduce respondent burden and any potential for confusion when more than one attribute is evaluated for a given AE. Conditional branching should be employed for electronic administration of PRO-CTCAE symptom terms that have two or more items. The logic branches from frequency, then to severity, then to interference. For example, if frequency is greater than never, the severity question is posed, and if severity greater than none, the interference question is posed [31]. Further, adding a “not applicable” option would introduce other difficulties since such a response choice is difficult to interpret as a meaningful response to a symptom-focused PRO item.

The primary limitation to the generalizability of our results is that, despite our best efforts, less than 10% of the study sample had low educational attainment (defined as less than high school graduate or the nearest equivalent). The sample, however, did include a large group of older adults, as well as a wide range of cancer diagnoses and treatment types.

Additional research is also needed to quantitatively evaluate the measurement properties of the Dutch language version of the PRO-CTCAE™ including construct validity, test-retest reliability and responsiveness to change over time. Additional research is needed to evaluate the Dutch language version of the PRO-CTCAE with lower literacy participants and when administering PRO-CTCAE via an interview, as compared to independent self-report. These knowledge gaps may be efficiently addressed by adding such psychometric research aims to future clinical trials and observational studies that include PRO-CTCAE as an outcome measure.

## Conclusion

The availability of the Dutch language version of the PRO-CTCAE item library will facilitate patient reporting of symptomatic AEs in the Netherlands and Dutch-speaking Belgium. This new measure also supports the global trend towards routine inclusion of the patient’s perspective in the evaluation of cancer treatments in both clinical trials and comparative effectiveness research [6, 32, 33].

## Supplementary information

**Additional file 1 Table S1**. Frequency table of participants debriefed by PRO-CTCAE symptom term and proportions endorsing difficulties with comprehension or judgement in Round 1.

## Data Availability

The datasets generated and analyzed during the current study are not publicly available because individual privacy could be compromised, but aggregated de-identified data are available from the corresponding author by reasonable request.

## Abbreviations

AE: Adverse Event
CTCAE: Common Terminology Criteria for Adverse Events
ISPOR: International Society for Pharmacoeconomic and Outcomes Research
NCI: United States National Cancer Institute
PROMs: Patient-Reported outcome measures
PRO-CTCAE™: Patient-Reported Outcomes version of the Common Terminology Criteria for Adverse Events™

## References

[1] Atkinson TM, Li Y, Coffey CW, Sit L, Shaw M, Lavene D (2012). Reliability of adverse symptom event reporting by clinicians. Quality of Life Research.

[2] Flores LT, Bennett AV, Law EB, Hajj C, Griffith MP, Goodman KA (2012). Patient-reported outcomes vs. clinician symptom reporting during chemoradiation for rectal cancer. Gastrointestinal Cancer Research.

[3] Basch E, Jia X, Heller G, Barz A, Sit L, Fruscione M (2009). Adverse symptom event reporting by patients vs clinicians: Relationships with clinical outcomes. Journal of the National Cancer Institute.

[4] Atkinson TM, Ryan SJ, Bennett AV, Stover AM, Saracino RM, Rogak LJ (2016). The association between clinician-based common terminology criteria for adverse events (CTCAE) and patient-reported outcomes (PRO): A systematic review. Supportive Care in Cancer.

[5] Denis F, Basch EM, Lethrosne C, Pourel N, Molinier O, Pointreau Y (2018). Randomized trial comparing a web-mediated follow-up via patient-reported outcomes (PRO) vs. routine surveillance in lung cancer patients: final results. Journal of Clinical Oncology.

[6] Basch E, Deal AM, Dueck AC, Scher HI, Kris MG, Hudis C, Schrag D (2017). Overall survival results of a trial assessing patient-reported outcomes for symptom monitoring during routine cancer treatment. Journal of the American Medical Association.

[7] Fromme EK, Eilers KM, Mori M, Hsieh YC, Beer TM (2004). How accurate is clinician reporting of chemotherapy adverse effects? A comparison with patient-reported symptoms from the quality-of-life questionnaire C30. Journal of Clinical Oncology.

[8] Basch E, Wood WA, Schrag D, Sima CS, Shaw M, Rogak LJ (2016). Feasibility and clinical impact of sharing patient-reported symptom toxicities and performance status with clinical investigators during a phase 2 cancer treatment trial. Clinical Trials.

[9] Basch E, Pugh SL, Dueck AC, Mitchell SA, Berk L, Fogh S (2017). Feasibility of patient reporting of symptomatic adverse events via the patient-reported outcomes version of the common terminology criteria for adverse events (PRO-CTCAE) in a chemoradiotherapy cooperative group multicenter clinical trial. International Journal of Radiation Oncology, Biology, Physics.

[10] Kluetz PG, Slagle A, Papadopoulos EJ, Johnson LL, Donoghue M, Kwitkowski VE (2016). Focusing on core patient-reported outcomes in cancer clinical trials: Symptomatic adverse events, physical function, and disease-related symptoms. Journal of Clinical Cancer Research.

[11] Basch, E., Reeve, B. B., Mitchell, S. A., Clauser, S. B., Minasian, L. M., Dueck, A. C., et al. (2014). Development of the National Cancer Institute's patient-reported outcomes version of the common terminology criteria for adverse events (PRO-CTCAE). *Journal of the National Cancer Institute*, *106*. 10.1093/jnci/dju244. 10.1093/jnci/dju244 PMC4200059 25265940

[12] Basch E, Reeve BB, Mitchell SA, Clauser SB, Minasian LM, Dueck AC (2014). Development of the National Cancer Institute’s patient-reported outcomes version of the common terminology criteria for adverse events (PRO-CTCAE). Journal of the National Cancer Institute.

[13] Dueck AC, Mendoza TR, Mitchell SA, Reeve BB, Castro KM, Rogak LJ (2015). Validity and reliability of the US National Cancer Institute's Patient-Reported Outcomes version of the Common Terminology Criteria for Adverse Events (PRO-CTCAE). Journal of the American Medical Association Oncology.

[14] Hay JL, Atkinson TM, Reeve BB, Mitchell SA, Mendoza TR, Willis G (2014). Cognitive interviewing of the US National Cancer Institute’s patient-reported outcomes version of the common terminology criteria for adverse events (PRO-CTCAE). Quality of Life Research.

[15] Bæksted C, Nissen A, Pappot H, Bidstrup PE, Mitchell SA, Basch E (2016). Danish translation and linguistic validation of the US National Cancer Institute's patient-reported outcomes version of the common terminology criteria for adverse events (PRO-CTCAE). Journal of Pain and Symptom Management.

[16] Kirsch M, Mitchell SA, Dobbels F, Stussi G, Basch E, Halter JP, De Geest S (2015). Linguistic and content validation of a German-language PRO-CTCAE-based patient-reported outcomes instrument to evaluate the late effect symptom experience after allogeneic hematopoietic stem cell transplantation. European Journal of Oncology Nursing.

[17] Arnold B, Mitchell SA, Lent L, Mendoza TR, Rogak LJ, Barragán NM (2016). Linguistic validation of the Spanish version of the National Cancer Institute’s patient-reported outcomes version of the common terminology criteria for adverse events (PRO-CTCAE). Supportive Care in Cancer.

[18] Yamaguchi T, Iwase S, Kuroda Y, Yamamoto D, Tsubota Y, Goto Y (2012). Introduction and current status of the development program for a Japanese version of the NCI PRO-CTCAE. Annals of Oncology.

[19] Wild D, Grove A, Martin M, Eremenco S, McElroy S, Verjee-Lorenz A, Erikson P (2005). Principles of good practice for the translation and cultural adaptation process for patient-reported outcomes (PRO) measures: Report of the ISPOR task force for translation and cultural adaptation. Value in Health.

[20] Reeve BB, Mitchell SA, Dueck AC, Basch E, Cella D, Reilly CM (2014). Recommended patient-reported core set of symptoms to measure in adult cancer treatment trials. Journal of the National Cancer Institute.

[21] Willis, G. B. (2004). *Cognitive interviewing: A tool for improving questionnaire design*. California: Sage Publications.

[22] Willis, G. B., Reeve, B. B, & Barofsky, I. (2004). The use of cognitive interviewing techniques in quality of life and patient-reported outcomes measurement. In Lipscomb, J., C.C. Gotay, C.C., and C. Snyder (Eds.), *Outcomes assessment in cancer: findings and recommendations of the cancer outcomes measurement working group*, pp. 610–622. Cambridge University Press.

[23] Miyaji T, Iioka Y, Kuroda Y, Yamamoto D, Iwase S, Goto Y (2017). Japanese translation and linguistic validation of the US National Cancer Institute’s Patient-Reported Outcomes version of the Common Terminology Criteria for Adverse Events (PRO-CTCAE). Journal of Patient-Reported Outcomes.

[24] Cho J, Yoon J, Kim Y, Oh D, Kim SJ, Ahn J (2019). Linguistic validation of the US National Cancer Institute’s Patient-Reported Outcomes Version of the Common Terminology Criteria for Adverse Events in Korean. Journal of Global Oncology.

[25] Terwee C, Roorda L, De Vet H, Dekker J, Westhovens R, Van Leeuwen J (2014). Dutch–Flemish translation of 17 item banks from the patient-reported outcomes measurement information system (PROMIS). Quality of Life Research.

[26] Sajobi TT, Brahmbatt R, Lix LM, Zumbo BD, Sawatzky RJ (2018). Scoping review of response shift methods: current reporting practices and recommendations. Quality of Life Research.

[27] Schwartz CE, Sprangers MA (2000). Methodological approaches for assessing response shift in longitudinal health-related quality-of-life research. Social Science and Medicine.

[28] Chung AE, Shoenbill K, Mitchell SA, Dueck AC, Schrag D, Bruner DW (2019). Patient free text reporting of symptomatic adverse events in cancer clinical research using the National Cancer Institute’s Patient-Reported Outcomes version of the Common Terminology Criteria for Adverse Events (PRO-CTCAE). Journal of the American Medical Informatics Association.

[29] Kluetz PG, Chingos DT, Basch EM, Mitchell SA (2016). Patient-reported outcomes in cancer clinical trials: Measuring symptomatic adverse events with the National Cancer Institute’s Patient-Reported Outcomes version of the Common Terminology Criteria for Adverse Events (PRO-CTCAE). American Society of Clinical Oncology Educational Book.

[30] Mendoza TR, Dueck AC, Bennett AV, Mitchell SA, Reeve BB, Atkinson TM (2017). Evaluation of different recall periods for the US National Cancer Institute’s PRO-CTCAE. Clinical Trials.

[31] United States National Cancer Institute (2019). Patient-reported outcomes version of the Common Terminology Criteria for Adverse Events (PRO-CTCAE™).

[32] Wilson MK, Collyar D, Chingos DT, Friedlander M, Ho TW, Karakasis K (2015). Outcomes and endpoints in cancer trials: bridging the divide. The Lancet Oncology.

[33] Basch E, Abernethy AP, Mullins CD, Reeve BB, Smith ML, Coons SJ (2012). Recommendations for incorporating patient-reported outcomes into clinical comparative effectiveness research in adult oncology. Journal of Clinical Oncology.

