# Supplementary material for: Dutch translation and linguistic validation of the U.S. National Cancer Institute’s Patient-Reported Outcomes version of the Common Terminology Criteria for Adverse Events (PRO-CTCAE™)
Source: J Patient Rep Outcomes. 2020 Oct 6;4:81. doi: 10.1186/s41687-020-00249-y (PMC7538479; doi:10.1186/s41687-020-00249-y)
Supplement: Supplementary file 1 — Additional file 1 Table S1. Frequency table of participants debriefed by PRO-CTCAE symptom term and proportions endorsing difficulties with comprehension or judgement in Round 1. [file 41687_2020_249_MOESM1_ESM.docx]

**Supplementary table 1** : *Frequency table of participants debriefed by PRO-CTCAE symptom term and proportions endorsing difficulties with comprehension or judgement in Round 1*

|  | ***Netherlands (N=60)*** | | ***Belgium***  ***(N=40)*** | | ***Pooled Sample***  ***(N=100)*** | |
| --- | --- | --- | --- | --- | --- | --- |
| **PRO-CTCAE SYMPTOM TERMS AND ATTRIBUTES** | **n*** | ***n^ǂ^/% reporting difficult to comprehend or judge*** | **n*** | ***n/% reporting difficult to comprehend or judge*** | **n*** | ***n/% found difficult to comprehend or judge*** |
| Attribute: Frequency | 60 | 1/60 (2) | 10 | 3/10 (30) | *70* | *4/70 (6)* |
| Attribute: Severity | 60 | 5/60 (8) | 10 | 0/10 (0) | *70* | *5/70 (7)* |
| Attribute: Interference | 60 | 6/60 (10) | 10 | 0/10 (0) | *70* | *6/70 (9)* |
| Concept: At its worst | 60 | 8/60 (13) | 10 | 1/10 (0) | *70* | *9/70 (13)* |
| **Oral** | | | | | | |
| Dry mouth | 14 | 0/14 (0) | 7 | 0/7 (0) | *21* | *0/21* (0) |
| Difficulty swallowing | 14 | 0/14 (0) | 7 | 0/7 (0) | *21* | *0/21* (0) |
| Mouth or throat sores | 60 | 4/60(10) | 30 | 0/30 (0) | *90* | *4/90 (4)* |
| Cracking at the corners of the mouth | 16 | 0/16 (0) | 8 | 0/8 (0) | *24* | *0/24* (0) |
| Voice changes | 16 | 1/16(6) | 7 | 0/7 (0) | *23* | *1/23 (4)* |
| Hoarse voice | 16 | 1/16 (6) | 8 | 0/8 (0) | *24* | *1/24 (4)* |
| **Gastro-Intestinal** | | | | | | |
| Taste changes | 16 | 0/16 (0) | 8 | 0/8 (0) | *24* | *0/24* (0) |
| Decreased appetite | 60 | 0/60 (0) | 10 | 0/10 | *70* | *0/70* (0) |
| Nausea | 60 | 0/60 (0) | 10 | 0/10 (0) | *70* | *0/70* (0) |
| Vomiting | 60 | 0/60 (0) | 10 | 0/10 (0) | *70* | *0/70* (0) |
| Heartburn | 16 | 0/16 (0) | 8 | 0/8 (0) | *24* | *0/24* (0) |
| Gas | 16 | 1/16 (0) | 8 | 0/8 (0) | *24* | *1/24 (4)* |
| Bloating | 14 | 0/14 (0) | 8 | 0/8 (0) | *22* | *0/22* (0) |
| Hiccups | 14 | 1/14 (7) | 7 | 0/7 (0) | *21* | *1/21 (5)* |
| Constipation | 60 | 1/60 (2) | 8 | 0/8 (0) | *68* | *1/68 (1)* |
| Loose or watery stools (diarrhea) | 60 | 0/60 (0) | 10 | 0/10 (0) | *70* | *0/70* (0) |
| Pain in the abdomen (belly area) | 14 | 0/14 (0) | 7 | 0/7 (0) | *21* | *0/21* (0) |
| Loss of control of bowel movements | 16 | 1/16 (6) | 8 | 0/8 (0) | *24* | *1/24 (4)* |
| **Respiratory** | | | | | | |
| Shortness of breath | 60 | 1/60 (2) | 30 | 0/30 (0) | *90* | *1/90 (1)* |
| Cough | 14 | 0/14 (0) | 7 | 0/7 (0) | *21* | *0/21 (0)* |
| Wheezing (whistling noise in the chest with breathing) | 15 | 0/15 (0) | 8 | 0/8 (0) | *23* | *0/23 (0)* |
| **Cardio/Circulatory** | | | | | | |
| Arm or leg swelling | 30 | 1/30 (3) | 30 | 0/30 (0) | *60* | *½* |
| Pounding or racing heartbeat (palpitations) | 16 | 0/16 (0) | 8 | 0/8 (0) | *24* | *0/0* |
| **Cutaneous** | | | | | | |
| Rash | 60 | 3/60 (5) | 10 | 0/10 (0) | *70* | *3/70 (4)* |
| Dry skin | 16 | 0/0 (16) | 8 | 0/8 (0) | *24* | *0/24* (0) |
| Acne or pimples on the face or chest | 14 | 0/14 (0) | 7 | 0/7 (0) | *21* | *0/21* (0) |
| Hair loss | 14 | 0/14 (0) | 7 | 0/7 (0) | *21* | *0/21* (0) |
| Itchy skin | 15 | 1/15 (7) | 8 | 0/8 (0) | *23* | *1/23 (4)* |
| Hives | 16 | 3/16 (19) | 8 | 0/8 (0) | *24* | *3/24 (13)* |
| Hand-foot syndrome | 16 | 3/16 (19) | 8 | 0/8 (0) | *24* | *3/24 (13)* |
| Lose any fingernails or toenails | 14 | 0/14 (0) | 7 | 0/7 (0) | *21* | *0/21 (0)* |
| Nail ridging | 14 | 2/14 (14) | 7 | 0/7 (0) | *21* | *2/21 (10)* |
| Nail discoloration | 14 | 0/14 (0) | 7 | 0/7 (0) | *21* | *0/21* (0) |
| Increased skin sensitivity to sunlight | 14 | 0/14 (0) | 7 | 0/7 (0) | *21* | *0/21* (0) |
| Bed/pressure sores | 14 | 0/14 (0) | 7 | 0/7 (0) | *21* | *0/21* (0) |
| Skin burns from radiation | 15 | 0/15 (0) | 8 | 0/7 (0) | *23* | *0/23* (0) |
| Unusual darkening of the skin | 16 | 1/16 (6) | 8 | 0/8 (0) | *24* | *1/24 (4)* |
| Stretch marks | 16 | 3/16 (19) | 8 | 0/8 (0) | *24* | *3/24 (13)* |
| **Neurological** | | | | | | |
| Dizziness | 16 | 0/16 (0) | 7 | 0/7 (0) | *23* | *0/23 (0)* |
| Numbness or tingling in your hands or feet | 60 | 0/60 (0) | 30 | 0/30 (0) | *90* | *0/90 (0)* |
| **Visual/Perceptual** | | | | | | |
| Blurry vision | 16 | 1/16 (6) | 8 | 0/8 (0) | *24* | *1/24 (4)* |
| Flashing lights | 16 | 1/16 (6) | 8 | 0/8 (0) | *24* | *1/24 (4)* |
| Visual floaters | 16 | 2/16 (13) | 8 | 0/8 (0) | *24* | *2/24 (8)* |
| Watery eyes (tearing) | 16 | 1/16 (6) | 8 | 0/8 (0) | *24* | *1/24 (4)* |
| Ringing in ears | 14 | 1/14 (7) | 7 | 0/7 (0) | *21* | *1/21 (5)* |
| **Attention/Memory** | | | | | | |
| Problems with concentration | 30 | 2/30 (7) | 30 | 0/30 (0) | *60* | *2/60 (3)* |
| Problems with memory | 29 | 0/29 (0) | 30 | 0/30 (0) | *59* | *0/59 (0)* |
| **Pain** | | | | | | |
| Pain | 60 | 4/60 (7) | 30 | 0/30 (0) | *90* | *4/90 (4)* |
| Headache | 28 | 0/28 (0) | 30 | 0/30 (0) | *58* | *0/54 (0)* |
| Muscle pain | 16 | 0/16 (0) | 8 | 0/8 (0) | *24* | *0/24 (0)* |
| Aching joints (such as elbows, knees, shoulders) | 15 | 0/15 (0) | 8 | 0/8 (0) | *23* | *0/23 (0)* |
| **Sleep/Wake** | | | | | | |
| Insomnia (including difficulty falling asleep, staying asleep, or waking up early) | 60 | 5/60 (8) | 30 | 0/30 (0) | *90* | *5/90 (6)* |
| Fatigue, tiredness, or lack of energy | 60 | 3/60 (5) | 10 | 0/10 (0) | *70* | *3/70 (4)* |
| **Mood** | | | | | | |
| Anxiety | 60 | 7/60 (12) | 30 | 0/30 (0) | *90* | *7/90 (8)* |
| Discouraged | 16 | 0/16 (0) | 30 | 0/30 (0) | *45* | *0/45 (0)* |
| Sad or unhappy feelings | 60 | 4/60 (7) | 30 | 0/30 (0) | *90* | *4/90 (4)* |
| **Gynecologic/Urinary** | | | | | | |
| Irregular menstrual periods/vaginal bleeding | 9 | 0/9 (0) | 8 | 0/8 (0) | *17* | *0/17 (0)* |
| Miss an expected menstrual period | 9 | 0/9 (0) | 8 | 0/8 (0) | *17* | *0/17 (0)* |
| Unusual vaginal discharge | 8 | 0/8 (0) | 12 | 0/12 (0) | *20* | *0/20 (0)* |
| Vaginal dryness | 9 | 2/9 (22) | 12 | 0/12 (0) | *21* | *2/21 (10)* |
| Pain or burning with urination | 16 | 0/16(0) | 8 | 0/8 (0) | *24* | *0/24 (0)* |
| Sudden urges to urinate | 14 | 1/14 (7) | 7 | 0/7 (0) | *21* | *1/21 (21)* |
| Urinary frequency | 14 | 0/14 (0) | 7 | 0/7 (0) | *21* | *0/21 (0)* |
| Change in usual urine color | 14 | 1/14 (7) | 7 | 0/7 (0) | *21* | *1/21 (5)* |
| Loss of control of urine (leakage) | 14 | 0/14 (0) | 7 | 0/7 (0) | *21* | *0/21 (0)* |
| **Sexual** | | | | | | |
| Difficulty getting or keeping an erection | 7 | 3/7 (43) | 12 | 0/12 (0) | *19* | *3/19 (16)* |
| Ejaculation problems | 7 | 0/7 (0) | 12 | 0/12 (0) | *19* | *0/19 (0)* |
| Decreased libido | 16 | 2/16 (13) | 8 | 0/8 (0) | *24* | *2/24 (0)* |
| Took too long to have an orgasm or climax | 16 | 3/16 (21) | 7 | 0/7 (0) | *24* | *3/24 (8)* |
| Unable to have an orgasm or climax | 16 | 2/16 (13) | 8 | 0/8 (0) | *24* | *2/24 (8)* |
| Pain during vaginal sex | 8 | 0/8 (0) | 9 | 0/9 (0) | *17* | *0/17 (0)* |
| **Miscellaneous** | | | | | | |
| Breast area enlargement or tenderness | 14 | 0/14 (0) | 7 | 0/7 (0) | *21* | *0/21 (0)* |
| Bruise easily (black and blue marks) | 14 | 0/14 (0) | 7 | 0/7 (0) | *21* | *0/21 (0)* |
| Shivering or shaking chills | 14 | 1/14 (7) | 7 | 0/7 (0) | *21* | *1/21 (5)* |
| Increased sweating | 14 | 4/14 (29) | 7 | 0/7 (0) | *21* | *4/21 (19)* |
| Unexpected decrease in sweating | 16 | 0/16 (0) | 8 | 0/8 (0) | *24* | *0/24 (0)* |
| Hot flashes | 14 | 0/14 (0) | 7 | 0/7 (0) | *21* | *0/21 (0)* |
| Nosebleeds | 16 | 0/16 (0) | 8 | 0/8 (0) | *24* | *0/24 (0)* |
| Pain and swelling at injection site | 14 | 1/ 14 (7) | 7 | 0/7 (0) | *21* | *1/21 (5)* |
| Body odor | 14 | 1/14 (7) | 7 | 0/7 (0) | *21* | *1/21 (5)* |

**Note:** *n represents the of participants who were debriefed; ^ǂ^ratio of the number of participants who had difficulties with the symptom term and the number of participants who were debriefed; numbers in parentheses represent the proportion of study participants who had difficulties with the symptom term
